# Adamantyl Retinoid-Related Molecules Induce Apoptosis in Pancreatic Cancer Cells by Inhibiting IGF-1R and Wnt/**β**-Catenin Pathways

**DOI:** 10.1155/2012/796729

**Published:** 2012-04-05

**Authors:** Lulu Farhana, Marcia I. Dawson, Jayanta K. Das, Farhan Murshed, Zebin Xia, Timothy J. Hadden, James Hatfield, Joseph A. Fontana

**Affiliations:** ^1^John D Dingell VA MC, Wayne State University, Detroit, MI 48201, USA; ^2^Department of Oncology, Wayne State University, Detroit, MI 48201, USA; ^3^Karmanos Cancer Institute, Wayne State University, Detroit, MI 48201, USA; ^4^Sanford-Burnham Medical Research Institute, La Jolla, CA 92037, USA; ^5^Department of Neurobiology, Harvard University, Cambridge, MA 02138, USA

## Abstract

Pancreatic carcinoma has a dismal prognosis as it often presents as locally advanced or metastatic. We have found that exposure to adamantyl-substituted retinoid-related (ARR) compounds 3-Cl-AHPC and AHP3 resulted in growth inhibition and apoptosis induction in PANC-1, Capan-2, and MiaPaCa-2 pancreatic cancer cell lines. In addition, AHP3 and 3-Cl-AHPC inhibited growth and induced apoptosis in spheres derived from the CD44^+^/CD24^+^ (CD133^+^/EpCAM^+^) stem-like cell population isolated from the pancreatic cancer cell lines. 3-Cl-AHPC-induced apoptosis was preceded by decreasing expression of IGF-1R, cyclin D1, **β**-catenin, and activated Notch-1 in the pancreatic cancer cell lines. Decreased IGF-1R expression inhibited PANC-1 proliferation, enhanced 3-Cl-AHPC-mediated apoptosis, and significantly decreased sphere formation. 3-Cl-AHPC inhibited the Wnt/**β**-catenin pathway as indicated by decreased **β**-catenin nuclear localization and inhibited Wnt/**β**-catenin activation of transcription factor TCF/LEF. Knockdown of **β**-catenin using sh-RNA also induced apoptosis and inhibited growth in pancreatic cancer cells. Thus, 3-Cl-AHPC and AHP3 induce apoptosis in pancreatic cancer cells and cancer stem-like cells and may serve as an important potential therapeutic agent in the treatment of pancreatic cancer.

## 1. Introduction

 Pancreatic cancer is the fourth leading cause of cancer associated mortality. Approximately 50% of the patients present with locally advanced unresectable disease and 35% with metastatic disease [1]. In those patients who undergo resection, 75% develop a recurrence and succumb to metastatic pancreatic cancer [1]. The dismal prognosis of pancreatic cancer is further accentuated by its poor response to chemotherapy and to radiation therapy. Although treatment with gemcitabine has resulted in some improvement in the overall well-being of some of the patients, no chemotherapeutic regimen has had a significant impact on the survival of patient with metastatic disease with median survivals in the 4 to 6 month range. Thus the discovery of new therapeutic agents and approaches to patients with pancreatic cancer is of paramount importance.

 Examination of tumors has resulted in the observation that a subpopulation of the tumor cells possess the properties of stem cells, that is, cells that are capable of undergoing self-renewal and as well as generating the heterogeneous lineages of cancer cells that comprise the majority of the tumor [2]. Pancreatic cancer stem cells have been identified in both pancreatic carcinoma cell lines as well as pancreatic cancer tissue obtained from patients [1, 3, 4]. A major emphasis has now been placed on detecting the specific pathways that are required for the proliferation and maintenance of these stem cells in the hope of developing specific targeted therapies resulting in the death of these cells.

 We and others have shown that a unique class of compounds termed the adamantyl-substituted retinoid-related molecules (ARRs) (*E*)-4-[3-(1-adamantyl)-4-hydroxyphenyl]-3-chlorocinnamic acid (3-Cl-AHPC) and (*E*)-3-{2-[3-(1-adamantyl)-4-hydroxyphenyl]-5-pyrimidinyl}-2-propenoic acid (AHP3 or BI-2005) inhibits proliferation and induces apoptosis *in vitro* and *in vivo* of a number of malignant cell types [5–9]. In this paper, we demonstrate that the ARRs are not only capable of inhibiting the growth of pancreatic cancer cells *in vitro *but also inhibit the growth and induce apoptosis in the pancreatic stem-like cell population. Exposure to the ARR compounds AHP3 and 3-Cl-AHPC resulted in the inhibition of stem cell sphere formation as well as apoptosis induction in the stem-like cells. Apoptosis induction was preceded by marked inhibition of IGF-1R, cyclin D1, *β*-catenin, and Notch-1 expression in pancreatic cells, but only IGF-1R, cyclin D1, and *β*-catenin in the cancer stem-like cells. Decreased IGF-1R expression enhanced ARR apoptosis induction and inhibited pancreatic carcinoma growth and sphere formation. *β*-catenin knockdown inhibited TCF/LEF transcriptional activity and downregulated Wnt/*β*-catenin target genes. The subsequent enhanced apoptosis and inhibition of growth in pancreatic cells suggests that the inhibition of Wnt/*β*-catenin signaling pathway is important for 3-Cl-AHPC-mediated apoptosis.

## 2. Materials and Methods

### 2.1. Reagents

3-Cl-AHPC was synthesized as described [5, 6]. DMEM-F12 medium, fetal bovine serum (FBS), lipofectamine 2000, and Prolong antifade kit were purchased from Invitrogen (Carlsbad, CA). Antibodies and their sources were as follows: antibodies for flow cytometry, CD44-PE, CD24-FITC, anti-EpCAM-PerCP-Cy5.5, and anti-c-Myc antibody from BD Biosciences (San Jose, CA) and CD44-APC-Cy7 and CD24-APC from Biolegend (San Diego, CA). Anti-CD44, anti-IGF-1R*β*, anti-*β*-catenin, anti-caspase 3, anti-cleaved caspase-3, and activated anti-cleaved Notch-1 (Val 1744) were from Cell Signaling Technology (Boston, MA); anti-CD24 and anti-cyclin D1 antibodies from Santa Cruz Biotechnology (Santa Cruz, CA); CD133-PE and CD326-FITC (EpCAM) from Miltenyi Biotec Inc. (Auburn, CA), and anti-*α*-tubulin antibody from Oncogene Research Products (Boston, MA). Anti-mouse IgG-TRITC conjugate for CD44, anti-rabbit IgG-FITC conjugate for CD24 and 3-(4,5-dimethylthiazol-2yl)-2, 5-diphenyltetrazolium bromide (MTT), 4′,6-Diamidino-2-phenylindole dihydrochloride (DAPI), and puromycin were purchased from Sigma-Aldrich (St. Louise, MO).

### 2.2. Cell Culture

Human pancreatic carcinoma cell lines, PANC-1, Capan-2, AsPc-1, MiaCaPa-2, and COLO357 were obtained from the American Type Culture Collection (ATCC, Rockville, MD) and maintained in DMEM-F12 medium containing 10% FBS and 100 *μ*g/mL gentamycin.

### 2.3. Apoptosis and Growth Inhibition

Pancreatic cancer cell lines were treated with 1 *μ*M 3-Cl-AHPC and AHP3 for various indicated time. Apoptosis in cells was analyzed by flow cytometry using Annexin V-FITC binding together with propidium iodide (PI) staining (Annexin V-FITC apoptosis Detection Kit 1, BD Biosciences, San Diego, CA). Data acquisition was done on a FACS Calibur flow cytometer (BD) and analyzed with CellQuest software (BD Biosciences). 3-Cl-AHPC- and AHP3-mediated inhibition of cell growth were determined by 3-(4,5-dimethylthiazol-2yl)-2,5-diphenyltetrazolium bromide (MTT) assay. The cells were seeded on 96-well plates at a density of 4 × 10^4^ cells/well in a volume of 200 *μ*L culture medium. 1 *μ*M AHP3 and 3-Cl-AHPC in DMSO (final concentration 0.1%) were added to the cells for various times. 25 *μ*L/well of MTT (5 mg/mL) was added to the medium and incubated for 4 h. After discarding the medium, MTT precipitates were solubilized with 200 *μ*L DMSO and the plates read on a BioTeK Synergy HT (BioTeK Instrument Inc., Vermont) at an absorbance 570 nm. All experiments were performed in quadruplicate to determine means and standard deviations. Cell apoptosis was assessed using acridine orange/ethidium bromide staining as described [10]. The spheres were stained with acridine orange/ethidium bromide staining and immediately visualized and photographed with fluorescence microscope (OLYMPUS CKX41). For DAPI staining, the spheres were incubated with DAPI stain for 30 minutes at 37°C. Then spheres were visualized and photographed with a fluorescence microscope.

## 3. Fluorescence-Activated Cell Sorting (FACS) of CD44^+^/CD24^+^ Cells and Sphere Formation

### 3.1. Isolation of CD44^+^/CD24^+^Cells

Cells were grown to 70–80% confluence and then trypsinised and washed with sorting buffer (1 × PBS, 5% FCS). The cells were resuspended with 100 *μ*L sorting buffer and incubated with 15–20 *μ*L anti-CD133-PE, anti-EpCAM-PerCP-Cy5.5, anti-CD24-FITC, and anti-CD44-PE primary antibodies for 30 min at ice. The cells were washed and resuspended in 500 *μ*L of sorting buffer and sorted using flow cytometry FACSAria system (BD Immunocytochemistry Systems, Franklin lakes, NJ).

### 3.2. Sphere Formation

The sorted CD133^+^, CD44^+^/CD24^+^ EpCAM^+^, and CD44^+^/CD24^+^ cells using flow cytometry were suspended in serum-free stem cell medium containing DMEM/F12 (1 : 1) supplemented with B27 (Life Technologies, Gaithersburg, MD), 20 ng/mL EGF (Biomol International, Plymouth, PA), 20 ng/mL fibroblast growth factor (Biomol International, Plymouth, PA), and 100 *μ*g/mL gentamycin. Approximately 150–200 cells per well were seeded in an ultralow-attachment 96-well plate (Corning Inc, Lowell, MA). 3-Cl-AHPC and AHP3 were added the day after cells were plated or after 7 days of sphere formation. Spheres were photographed and measured utilizing an Olympus microscope (OLYMPUS CKX41) and Olympus microscope digital camera with DP2-BSW software (Olympus soft imaging solutions GmbH, Germany).

### 3.3. Western Blots

Cells were extracted with lysis buffer containing 25 mM Tris-Cl buffer (pH 8.0), 150 mM NaCl, 0.2% nondiet P-40, 10% glycerol 10 mM NaF, 8 mM *β*-glycerophosphate, 0.2 mM Na_3_VO_4_, 1 mM DTT, and 10 *μ*L/mL protease inhibitor cocktail (Sigma Aldrich, St. Louise, MO), and Western blots were performed as we previously described [11].

### 3.4. Immunofluorescence

Approximately 150 spheres were fixed with 4% paraformaldehyde in 1% Triton X-100, washed in PBS, dehydrated in methanol (25%, 50%, 75% 95%, and 100%), and then rehydrated in descending percentage of methanol and washed in PBS. Spheroids were incubated in 3% normal goat serum (Vector Lab, Burlingame, CA) at 4°C for 24 h and washed in phosphate-buffered saline with 0.5% Tween 20 (PBST). Then spheres were incubated with primary antibodies anti-CD44 and anti-CD24 for 48 h at 4°C, washed in PBST, and incubated with anti-mouse IgG-TRITC conjugate for CD44 and CD133 and anti-rabbit IgG-FITC conjugate for CD24 and CD326 (EpCAM) for 24 h. Spheres were mounted in 8 chambered slides and fluorescence staining analyzed. Spheres grown in 96-well ultralow-attachment plates were incubated with DAPI at 37°C for 30 minutes to assess DAPI staining. For *β*-catenin immunostaining, PANC-1cells were grown in eight chambered slide and then treated with 3-Cl-AHPC for 24 h. Cells were blocked with 3% normal goat serum at 4°C for 1 h and then incubated with anti-*β*-catenin antibody for overnight at 4°C. After washing with 1XPBS, cells were incubated with anti-rabbit IgG-FITC conjugate antibody for 2 h. Cells were washed with PBS and then placed on cover slips with prolong gold antifade reagent (Cell Signaling Technology, Boston, MA).

### 3.5. Sphere Block Preparation and In Situ Sphere Cell Death Detection

DMSO (vehicle) and 3-Cl-AHPC-treated spheres were centrifuged at 1000 rpm for 5 minutes, washed in PBS, 22% bovine serum albumin added to the spheres pellet, 95% ethanol placed on the spheres pellet, and the pellet allowed to harden for 30 minutes. Neutral buffered formalin (10%) was added to fix the cell pellet for at least 2 h and the spheres were then placed in a labeled plastic tissue embedding cassette containing 10% neutral buffered formalin overnight. The spheres were processed in a Sakura Tissue-Tek Processor for overnight dehydration in graded ethanol, clearing in xylene and infiltration with paraffin. The spheres were placed in a 4 *μ*m embedding mold for final paraffin embedding.

The TUNEL assay was performed using the *In Situ* Cell Death Detection kit, POD (Roche-Applied-Science, Mannheim, Germany), according to the manufacturer's instructions. The paraffin embedding spheres were deparaffinized and rehydrated; then tissues sections were incubated with proteinase K solution (10–20 *μ*g/mL) for 30 min. Tissues were then rinsed twice in PBS and reacted with 50 *μ*L of the TUNEL reaction mixture at room temperature for 60 min in a dark, humidified chamber. Sections were again rinsed in PBS and incubated for 30 min with 50 *μ*L of the Converter-POD (Roche-Applied-Science) and followed by 3-amino-9-ethylcarbazole (AEC). Sections were then counterstained with hematoxylin. As negative controls, corresponding sections were treated in the same way without terminal deoxynucleotidyl transferase.

### 3.6. shRNA Plasmids

Human GIPZ lentiviral shRNAmir expression vector GFP-tagged-pGIPZ-shRNA-IGF-1R was purchased from Open Biosystems (Thermo Scientific, Huntsville, AL). shRNA-IGF-1R expression vectors were stably transfected into PANC-1 cell lines using lipofectamine 2000. Stable cell lines were selected with puromycin. The scrambled sequence shRNA-vector was used as a control. pGIPZ-shRNA expression vector clone ID V2LSH-20147, V2LSH-131072, V3LSH-377850, V3LSH-377852, and V3LSH-377849 inhibited IGF-1R expression more effectively in PANC-1 cells than other clones from a set of eight tested clones.

sh-RNA-*β*-catenin-pSIREN-RetroQ expression vectors were constructed according to the manufacturer's instructions (Clontech, CA). The gene silencing target sequences were from the coding sequence of the PubMed Accession number NM_001904.3, and sh-RNA sequences 5′-CCATggAACCAgACAgAAA-3′ (catenin-KD-1) and 5′-ggATgTggATACCTCCCAAg-3′ (catenin-KD2) were synthesized from Integrated DNA technology Inc. (Coralville, IA). sh-*β*-catenin sequences were used for directional cloning 5′-*Bam*H I and 3 *EcoR* I overhang nucleotide in pSIREN-RetroQ vector. shRNA regions in plasmid backbone were confirmed by sequencing. sh-*β*-catenin plasmids were used to transfect the cell lines utilizing the lipofectamine method. The stable cell lines were selected with puromycin, and sh-*β*-catenin knockdown cell lines were grown in presence of puromycin. Scramble sequence sh-vector was used as a control.

### 3.7. TCF/LEF-Luciferase Assay

In order to determine the activation of Wnt/*β*-catenin signaling, the transcription factor T-cell-factor/lymphoid-enhancing-factor- (TCF/LEF)-Luc reporter plasmid was used in PANC-1 cells. Cells were transduced with Cignal TCF/LEF-Luc reporter lentiviral plasmid (SA Biosciences, Frederick, MD) in presence of polybrene (8 *μ*g/mL) for 48 h and the cells were selected with puromycin (1 *μ*g/mL). TCF/LEF cells were treated with 3-Cl-AHPC for 24 h. Cells were harvested and analyzed for TCF/LEF activity using a luciferase assay kit (Promega-Biosciences, San Luis Obispo, CA) as followed by the instructions of manufacturer and the activity was measured on a BioTeK Synergy HT.

### 3.8. Statistical Analysis

All statistics were performed using VassarStats web statistical software (Richard Lowry, Poughkeepsie, NY, USA). One-way analysis of variance (ANOVA) was performed to detect any differences between groups of sphere control, 3-Cl-AHPC-treated spheres and AHP3-treated spheres. If the result of the ANOVA is significant (*P* < 0.01 versus control), pairwise comparisons between the groups were made by a post hoc test (Tukey's HSD procedure). The significance level was set at *P* < 0.01 versus control and *P* < 0.05 versus control. Square brackets were used in the figures to indicate treatments that are significantly different from the control.

## 4. Results

### 4.1. AHP3 and 3-Cl-AHPC Induction of Apoptosis in COLO357, PANC-1, AsPc-1, Capan-2, and MiaPaCa-2 Cells

**Figure 1:**
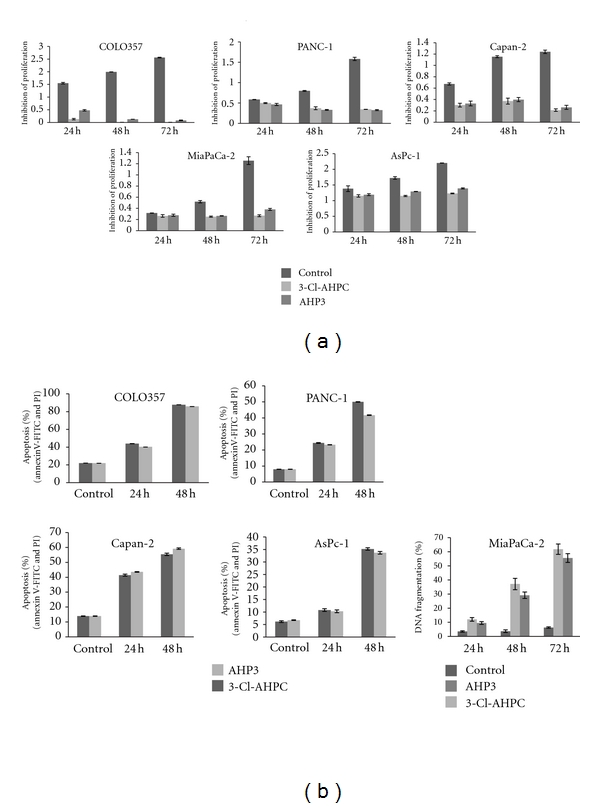
3-Cl-AHPC- and AHP3-mediated proliferation inhibition and apoptosis induction in pancreatic cancer cell lines. The cells were exposed to 1 *μ*M 3-Cl-AHPC or AHP3 for various times. (a) Proliferation inhibition was evaluated by MTT assay as described Section 2 and expressed as absorbance (OD) measured at 570 nm. The error bars represent the mean of three separate determinations ± the standard deviation (SD). (b) Induction of apoptosis in pancreatic cancer cells by 3-Cl-AHPC and AHP3. Cells were seeded at 1 × 10^4^ cells/mL and grown for 24 h and then exposed to 1 *μ*M AHP3 or 3-Cl-AHPC for indicated times. Induction of apoptosis and cell death was assessed using Annexin V-FITC labeling with propidium iodide (PI) staining in COLO357, PANC-1, Capan-2 AsPc-1, or acridine orange/ethidium bromide staining in MiaPaCa-2. The error bars represent the mean of three separate determinations ± the standard deviation (SD). All treated samples are significantly different from vehicle control.

AHP3 and 3-Cl-AHPC inhibited growth and induced apoptosis in Ras wild type COLO357 and Ras mutant PANC-1, AsPc-1, Capan-2, and MiaPaCA-2 pancreatic carcinoma cells. 1 *μ*M AHP3 or 3-Cl-AHPC resulted in the inhibition of proliferation (Figure 1(a)). There was an 80% inhibition COLO357 growth, 50 to 60% inhibition of PANC-1 growth, 70% inhibition of Capan-2 growth, and 60 to 70% inhibition of MiaPaCa-2 cells at 72 h by both 3-Cl-AHPC and AHP3. AsPc-1 cells demonstrated increased resistance to both 3-Cl-AHPC and AHP3 with only 47% and 25% growth inhibition by 3-Cl-AHPC and AHP3, respectively, at 72 h (Figure 1(a)).

Exposure of COLO357 cells to 1 *μ*M concentrations of 3-Cl-AHPC or AHP3 resulted in apoptosis induction of 80% of the cells at 48 h. Ras mutant cell lines displayed enhanced resistance to AHP3 and 3-Cl-AHPC-mediated apoptosis when compared to Ras wild type cell line COLO357 (Figure 1(b)). 3-Cl-AHPC and AHP3 exposure resulted in 22% apoptosis at 24 h and 50% and 48% apoptosis, respectively, at 48 h in PANC 1 cells (Figure 1(b)). Similar results were noted with Capan-2 cells with 40 and 50% apoptosis at 24 h and 48 h, respectively (Figure 1(b)). 3-Cl-AHPC and AHP3 exposure resulted in 35% and 40% apoptosis of AsPC-1 cells at 48 h, respectively (Figure 1(b)). Control MiaPaCa-2 cells showed 60% Annexin V-FITC positivity; however, these cells were not apoptotic by any other criteria and grew normally in culture. Therefore, acridine orange/ethidium bromide staining was utilized as we have previously described to assess DNA fragmentation and apoptosis [6]. Exposure to 3-Cl-AHPC and AHP3 resulted in 30% apoptosis at 48 h and 60% induction of apoptosis at 72 h and 96 h.

 Numerous studies have supported the concept that the stem cell population is responsible for the persistent resistance of cancer cells to chemotherapy as well as their metastatic behavior [12–15]. Previous studies have shown that pancreatic cancers and pancreatic cancer cell lines contain a small segment of cell population characterized by expression of CD133 or CD44/CD24/EpCAM positivity and which can be utilized to identify this cancer stem cell population [16–19]. While there appears to be significant overlap between CD133 positive and CD44^+^/CD24^+^ cells, this overlap appears to vary between different tumor samples with only 10 to 40% of the CD44^+^/CD24^+^ cells expressing CD133 [4]. The intriguing observation that both CD133 positive and CD44^+^/CD24^+^ cells have been found to be tumor initiating cells and possess many of the characteristics of cancer stem cells suggests that heterogeneity exists in the pancreatic cancer stem cell population.

**Figure 2:**
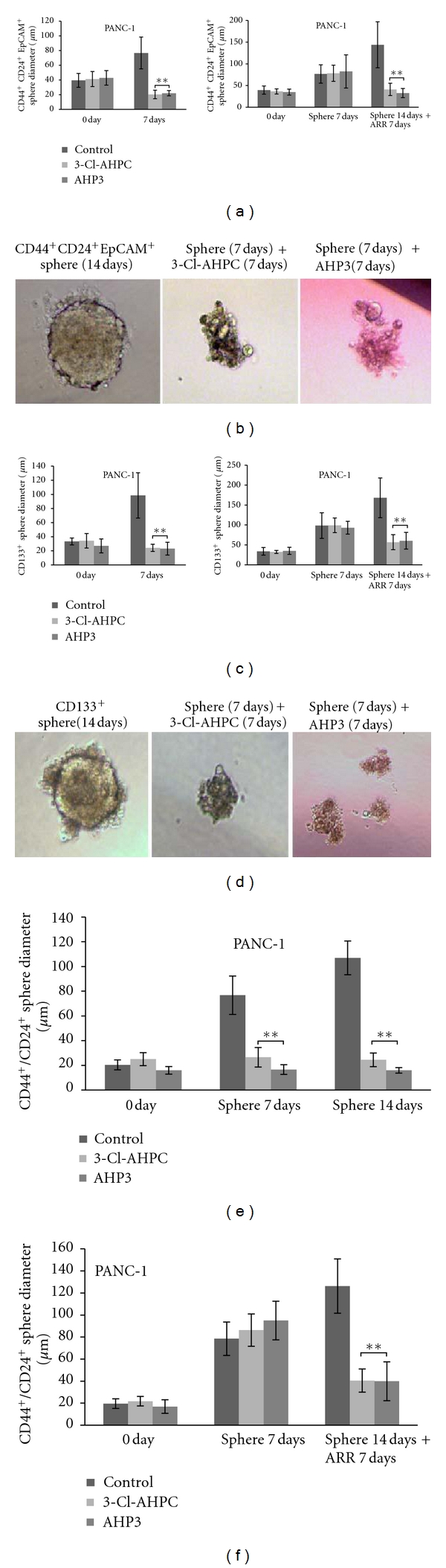
3-Cl-AHPC-mediated inhibition and degradation of pancreatic cancer stem cells spheres of CD133^+^, CD44^+^/CD24^+^/EpCAM^+^, and stem-like spheres of CD44^+^/CD24^+^ PANC-1 cells. ((a), (b) and (c), (d)) 3-Cl-AHPC and AHP3 exposure resulted in inhibition of CD44^+^/CD24^+^/EpCAM^+^ and CD133^+^cells growth and sphere formation and degradation of the derived spheres. ((e), (f)) AHP3 and 3-Cl-AHPC inhibited sphere formation and inhibition of growth and degradation of the CD44^+^/CD24^+^-derived spheres. For sphere formation, the CD44^+^/CD24^+^/EpCAM^+^, CD133^+^, and CD44^+^/CD24^+^ cells were sorted by flow cytometry and approximately 200–300 cells were seeded with B27 containing DMEM/F12 medium in 96-well low attachment plates and 1 *μ*M 3-Cl-AHPC or AHP3 added either the day after seeding or 7 days following sphere formation. The sizes of spheres were photographed and measured on a 100 *μ*m scale and magnification 400X using Olympus fluorescence microscope digital camera software and DP2-BSW software. The error bars represent the mean of 15 sphere determinations ± the standard deviation. ** was significantly different in comparison to control spheres. Data were analyzed by ANOVA, Tukey HSD test for multiple comparisons. **P < 0.01 versus control.

 Pancreatic cancer stem cells have been characterized by stem cell markers CD133^+^ and CD44^+^/CD24^+^/EpCAM^+^ (epithelial adhesion molecule)/ESA (epithelial specific antigen) [3, 4, 20]. To investigate the efficiency of ARR in inhibiting the growth of cancer stem cells, we examined the inhibition of sphere formation in CD44^+^/CD24^+^/EpCAM^+^ and CD133^+^ cancer stem cells. PANC-1 cells were sorted by flow cytometry to obtain CD133^+^ and CD44^+^/CD24^+^/EpCAM^+^expressing cells and cells were allowed to form spheres in the DMEM/F12-B27 medium for 7 days. 3-Cl-AHPC and AHP3 exposure completely inhibited sphere formation as well as addition of 3-Cl-AHPC or AHP3 on day 7 after sphere formation resulted in significant inhibition of sphere growth of CD133^+^ and CD44^+^/CD24^+^/EpCAM^+^ PANC-1 stem cells (Figures 2(a)–2(d)). Sphere formation in nonadherent conditions is a unique property of cancer stem cells in general [16]. Studies have demonstrated resistance of pancreatic stem cells to chemotherapy agents including gemcitabine [3, 12, 15]. We assessed the ability of ARRs to inhibit sphere formation from the sorted CD44^+^/CD24^+^ stem-like cells in B27 medium. 3-Cl-AHPC or AHP3 addition at the time of PANC-1 seeding in B27 medium completely inhibited sphere formation by the cells at day 7 and day 14 (Figure 2(e) and see Figure S1A in Supplementary Material available online at doi: 10.1155/2012/796729.), while addition of 3-Cl-AHPC or AHP3 on day 7 after sphere formation resulted in a 70% inhibition of sphere formation as well as degradation of the sphere cells at day 14 (Figure 2(f) and Supplementary Figure S1B). In order to determine the expression of CD44, CD24, EpCAM, and CD133 in CD44^+^/CD24^+^ sphere cells, we stained the spheres with fluorescent antibody for TRITC-conjugate-CD44 and CD133, and FITC-conjugate-CD24 and EpCAM. Utilizing confocal microscopy overlay, we confirmed that the PANC-1 spheres consisted of CD44^+^/CD24^+^ cells (Supplementary Figure S2A). These cells expressed not only CD44^+^/CD24^+^ but also CD133 as well as EpCAM (Supplementary Figure S2B).

**Figure 3:**
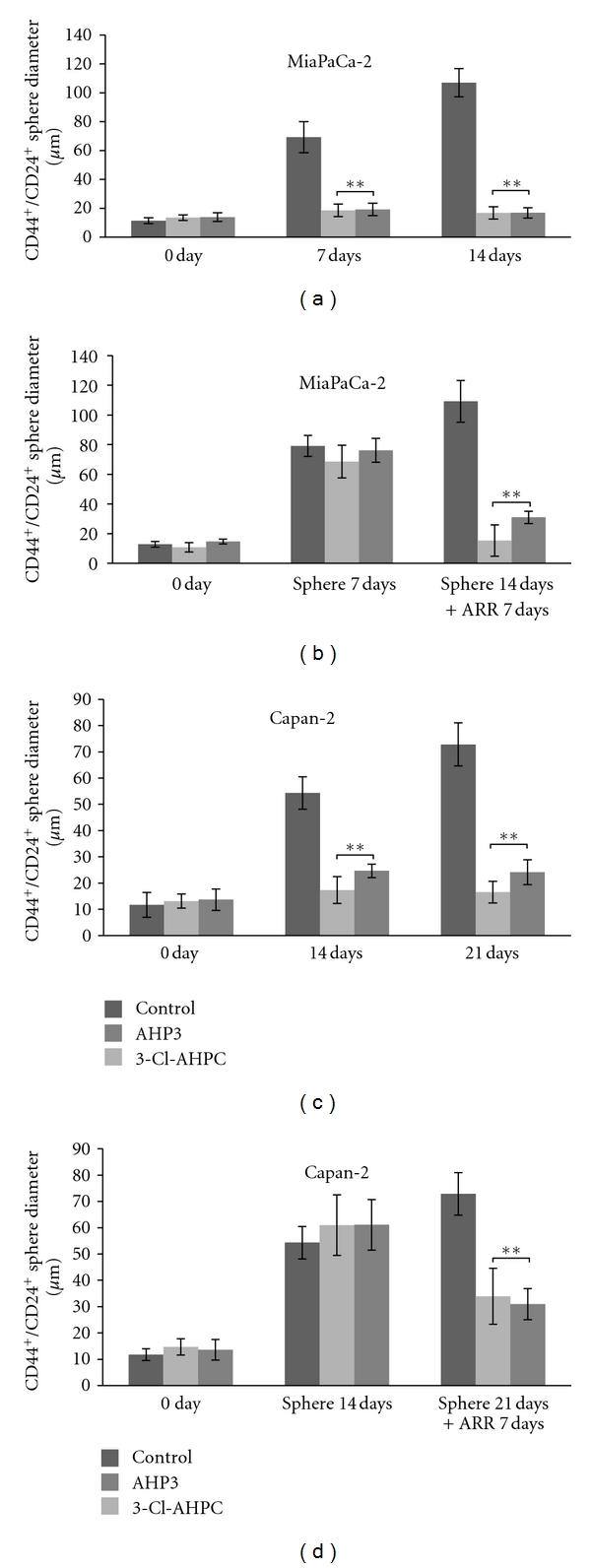
3-Cl-AHPC- (1 *μ*M) and AHP3- (1 *μ*M) mediated inhibition of CD44^+^/CD24^+^ stem-like cell sphere formation and degradation of spheres derived from MiaPaCa-2 and Capan-2 cell lines. 3-Cl-AHPC and AHP3 were added at the time cells were seeded ((a), (c)) or 7 days after cells sphere formation ((b), (d)). The ARR affect on sphere growth was assessed at days 7 and 14 (a), days 7 and 14 (b), days 14 and 21 (c), and days 14 and 21 (d), respectively. The error bars represent the mean of 15 sphere determinations ± the standard deviation. ** Was significantly different in comparison to control spheres. Data were analyzed by ANOVA, Tukey HSD test for multiple comparisons. **P < 0.01 versus control.

3-Cl-AHPC or AHP3 addition to the CD44^+^/CD24^+^ MiaPaCa-2 and Capan-2 cells at the time of seeding in the B27 medium also totally inhibited spheres formation (Figures 3(a) and 3(c)). Similarly, 3-Cl-AHPC or AHP3 exposure at day 7 after sphere formation taken place resulted in a 70% and 50% inhibition of sphere size in the MiaPaCa-2 and Capan-2 cells, respectively (Figures 3(b) and 3(d)).

**Figure 4:**
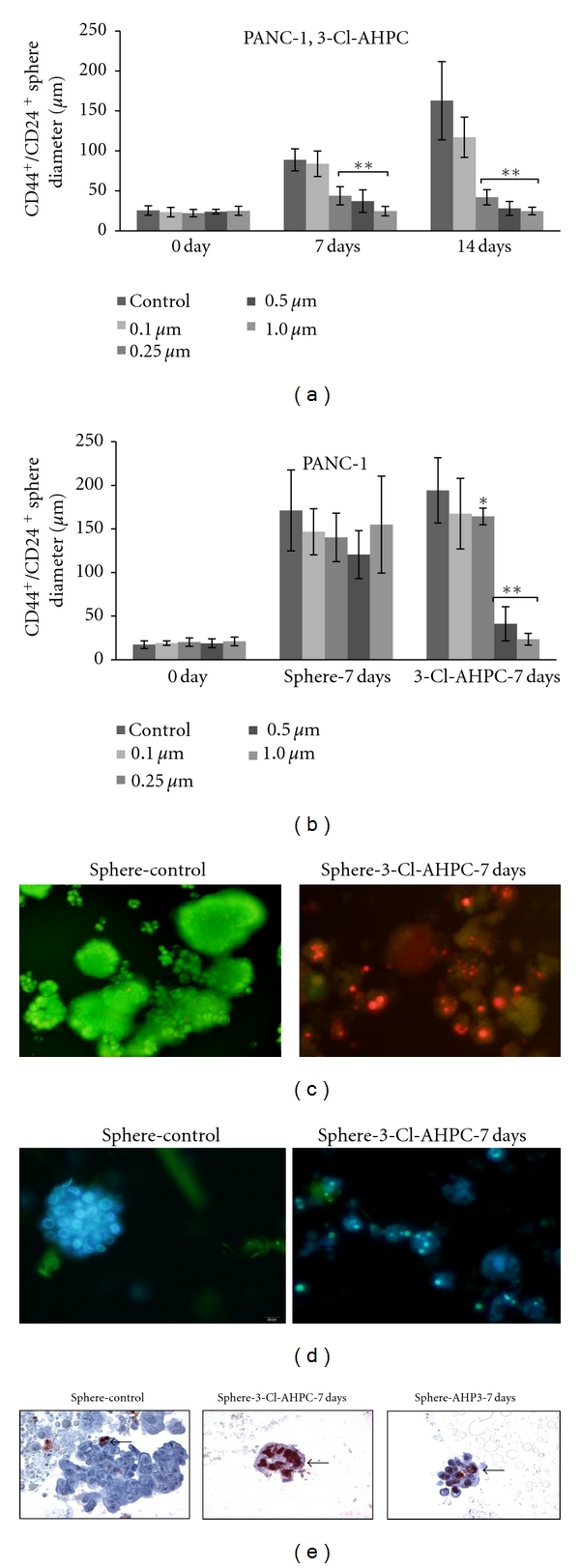
Dose-response effect of 3-Cl-AHPC on CD44^+^/CD24^+^ cells sphere formation and apoptosis in PANC-1 sphere cells. (a) Addition of 0.25, 0.5, and 1.0 *μ*M 3-Cl-AHPC added at time of cell seeding inhibited sphere formation at 7 and 14 days. (b) 0.5 and 1.0 *μ*M 3-Cl-AHPC inhibited sphere formation when added 7 days following sphere formation. (c) 1.0 *μ*M 3-Cl-AHPC induced apoptosis in CD44^+^/CD24^+^ sphere cells as indicated by nuclear fragmentation detected by acridine orange/ethidium bromide and (d) DAPI staining. Spheres were visualized and photographed utilizing a fluorescence microscope. (e) Apoptosis of sphere cells as demonstrated by TUNEL assay. CD44^+^/CD24^+^ spheres were treated with 1.0 *μ*M ARRs for 7 days (7D) after sphere formation. Details of slides preparation, visualization, antibodies utilized, and TUNNEL assay methodologies were as described in Section 2.

The 3-Cl-AHPC dose required to inhibit sphere formation by the PANC-1 cells was determined. The addition of 0.25, 0.5, or 1 *μ*M 3-Cl-AHPC to PANC-1 cells at the time of seeding in B27 medium resulted in more than a 50% inhibition of sphere formation (Figure 4(a)); 0.1 *μ*M did not inhibit sphere formation (Figure 4(a)). 3-Cl-AHPC concentrations of 0.5 and 1 *μ*M were required to reduce PANC-1 sphere size greater than 80% when added at day 7 after sphere formation (Figure 4(b)), resulting in disaggregation of the sphere and apoptosis induction of the cells, as documented by DNA fragmentation demonstrated by acridine orange/ethidium bromide, DAPI staining, and positive TUNEL staining of the spheres (Figures 4(c), 4(d), and 4(e)).

**Figure 5:**
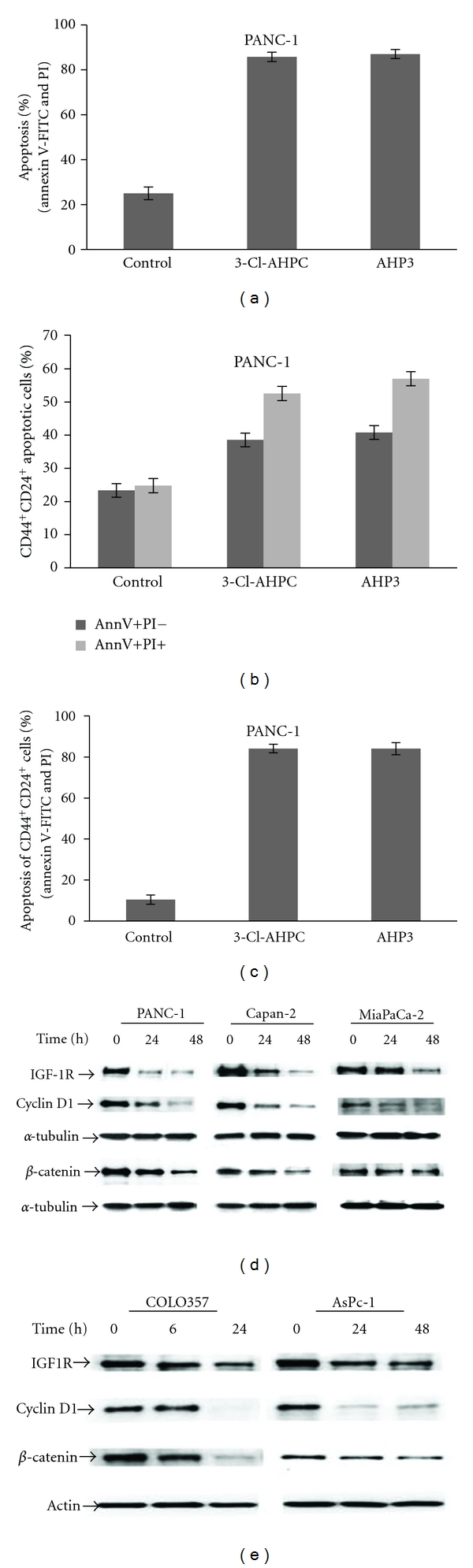
3-Cl-AHPC and AHP induced apoptosis in PANC-1 CD44^+^/CD24^+^ cells and 3-Cl-AHPC decreased expression of IGF-1R, cyclin D1, and *β*-catenin in pancreatic cancer cells. (a) Percentage of total apoptotic cells. (b) Percentage of CD44^+^/CD24^+^ cells in the early (Annexin V-FITC positive and PI negative) or late (Annexin V-FITC positive and PI positive) apoptotic cell populations. (c) Percentage of total CD44^+^/CD24^+^ apoptotic cells (Annexin V-FITC positive and PI positive). Cells were treated with 1.0 *μ*M 3-Cl-AHPC and AHP3 for 96 h. Antibody-conjugated markers CD44-APC-Cy7, CD24-APC, Annexin V-FITC, and PI were used to detect apoptotic and CD44^+^/CD24^+^ cells from the same samples. The error bars represent the mean of three separate determinations ± the standard deviation. ((d), (e)) IGF-1R, cyclin D1, and *β*-catenin expression decreased following 3-Cl-AHPC exposure in pancreatic cancer cells.

Further evidence of AHP3 and 3-Cl-AHPC-mediated apoptosis of CD44^+^/CD24^+^ PANC-1 cells was demonstrated by Annexin V-FITC and PI staining. 3-Cl-AHPC and AHP3 exposure of PANC-1 cells resulted in 80% apoptosis after 96 h treatment (Figure 5(a) and Supplementary Figure S2C). We sorted the 3-Cl-AHPC- and AHP3-treated PANC-1 cells for CD44^+^/CD24^+^cells and assessed the percentage of these cells that were apoptotic by determining Annexin V-FITC positive staining. Flow cytometry analysis showed that CD44^+^/CD24^+^-gated early apoptotic (Annexin V-FITC + and PI−) cells were 36% and 40% for the 3-Cl-AHPC- and AHP3-treated cells, respectively (Figure 5(b) and Supplementary Figure S2D, upper panel). Late apoptotic cells (Annexin V-FITC + and PI +) were 51% and 57% for the 3-Cl-AHPC- and AHP3-treated cells, respectively (Figure 5(b) and Supplementary Figure S2D, bottom panel). Thus, a total of 80% of the CD44^+^/CD24^+^ PANC-1 cell population were apoptotic after 96 h exposure to either 3-Cl-AHPC or AHP3 (Figure 5(c) and Supplementary Figure S2E).

The ability of AHP3 and 3-Cl-AHPC to induce apoptosis in CD44^−^/CD24^−^, CD44^+^/CD24^−^, CD44^−^/CD24^+^, and CD44^+^/CD24^+^ cells was also examined. These cell populations were isolated from PANC-1 cells and treated with 3-Cl-AHPC. The addition of 1 *μ*M 3-Cl-AHPC to the various cell populations resulted in the growth inhibition and the induction of apoptosis as indicated by DNA fragmentation (Supplementary Figures S3 and S4).

### 4.2. 3-Cl-AHPC Decreases Expression of IGF-1R, Cyclin D1, and *β*-Catenin in Pancreatic Cancer Cells

3-Cl-AHPC and AHP3 exposure on the expression of cyclin D1, *β*-catenin, and IGF-1R in the pancreatic cancer cells was assessed. Cyclin D1 is an important regulatory protein required in cell cycle progression, and overexpression has been associated with a poor prognosis in patients with pancreatic cancer [21]. Inhibition of cyclin D1 expression has been found to inhibit pancreatic cancer growth [21]. Abnormal expression of *β*-catenin was found to be associated with the development of metastatic pancreatic cancer as well as the upregulation of cyclin D1, c-Myc, and matrix-metalloproteinase-7 [22, 23]. IGF-1R and IGF-1 are overexpressed in human pancreatic tumors. IGF-1R signaling regulates proliferation, invasion, and angiogenic growth factor expression by pancreatic cancer cells [24–26].

3-Cl-AHPC exposure on pancreatic cancer cells decreased expression of IGF-1R, cyclin D1, and *β*-catenin prior to the inhibition of proliferation and the induction of apoptosis (Figures 5(d) and 5(e)). There was a 82%, 90%, 68%, and 31% inhibition of IGF-1R expression in the PANC-1, Capan-2, MiaPaCa-2, and AsPc-1cells, respectively, at 48 h following 3-Cl-AHPC exposure. 3-Cl-AHPC-mediated inhibition of cyclin D1 expression was 80%, 85%, 52%, and 69% in PANC-1, Capan-2, MiaPaCa-2, and AsPc-1cells, respectively, at 48 h (Figures 5(d) and 5(e), and Supplementary Figure S5A). The addition of 3-Cl-AHPC to the PANC-1, Capan-2, MiaPaCa-2, and AsPc-1 cells resulted in a decrease of 65%, 66%, 22%, and 43%, respectively, in *β*-catenin expression (Figures 5(a) and 5(e) and Supplementary Figure S5A). Similarly, 3-Cl-AHPC exposure in COLO357 cells decreased IGF-1R (43%), cyclin D1 (93%), and *β*-catenin (85%), respectively, at 24 h following 3-Cl-AHPC exposure (Figure 5(e) and Supplementary Figure S5A). AHP3 exposure in PANC-1 cells also decreased IGF-1R, cyclin D1, and *β*-catenin (Supplementary Figure  S5B). These results suggest that decrease of IGF-1R, cyclin D1, and *β*-catenin reflects a phenomenon general to ARR-mediated apoptosis induction in pancreatic cancer cells.

**Figure 6:**
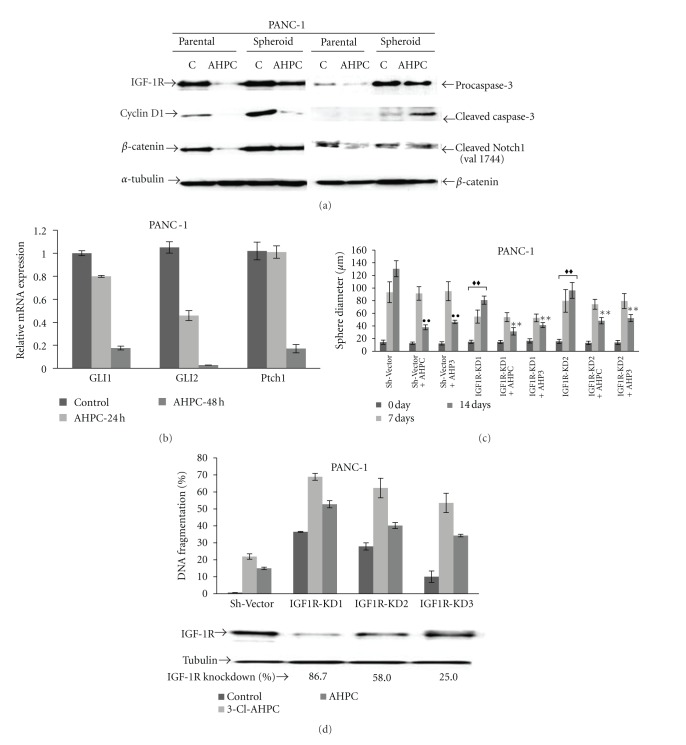
3-Cl-AHPC decreased expression of IGF-1R, cyclin D1, *β*-catenin, and cleaved Notch-1 and increased levels of cleaved-caspase-3 in CD44^+^/CD24^+^spheres. (a) IGF-1R, cyclin D1, and *β*-catenin expression decreased cleaved-caspase-3 increased with no change in Notch-1 protein levels in spheres following exposure to 3-Cl-AHPC. Pancreatic cancer cells and PANC-1 spheres were exposed to 1.0 *μ*M 3-Cl-AHPC for 7 days. Western blots were prepared as described in Materials and Methods. (b) mRNA expression of GLI1, GLI2, and Ptch1 in PANC-1 cells. Cells were grown in the presence of 1 *μ*M 3-Cl-AHPC or vehicle alone (control). (c) Knockdown of IGF-1R expression by sh-IGF-1R inhibited sphere formation and enhanced ARR inhibition of sphere formation. The error bars represent the mean of three separate determinations +/− the standard deviation. •• was significantly different between spheres comprised of sh-vector cells treated with vehicle and 3-Cl-AHPC or AHP3. ♦♦ was significantly different between spheres comprised of sh-vector and IGF-1R-KD1 or IGF-1R-KD 2 at 7 and 14 days, respectively. ** was significantly different in comparison between IGF-1R-KDl/IGF-1R-KD2 spheres (vehicle treated) and IGF-1R-KD1/IGF-1R-KD2 spheres treated with 3-Cl-AHPC or AHP3. Data were analyzed by ANOVA, Tukey HSD test for multiple comparisons, ♦♦, ••, and **P < 0.01. (d) Knockdown (KD) of IGF-1R enhanced AHP3- and 3-Cl-AHPC-mediated apoptosis in the PANC-1 cells and IGF-IR protein expression in IGF-1R knockdown cells. Apoptosis was assessed using acridine orange/ethidium bromide staining as described in Section 2.

A 58%, 95%, and 50% decrease in IGF-1R, cyclin D, and *β*-catenin expression was noted in the PANC-1 CD44^+^/CD24^+^spheres following exposure to 3-Cl-AHPC, respectively, followed by apoptosis (Figure 6(a) and Supplementary Figure S5C). Sphere apoptosis was supported by the observation of cleavage of caspase 3 following 3-Cl-AHPC exposure on PANC-1 sphere cells (Figure 6(a)).

Self-renewal of CSCs has been shown to be regulated by the Wnt/*β* catenin, Hedgehog, and Notch signaling pathways [27, 28]. Lee et al. [4] found that expression of sonic hedgehog transcripts was increased by 46-fold in the CD44/CD24/EpCAM positive cells derived from pancreatic cancer cells while there was only a 4-fold increase in the CD44/CD24/EpCAM negative population. 3-Cl-AHPC downregulated the GLI1, GLI2, and Ptch1 mRNA expression in the hedgehog pathway (Figure 6(b)) and decreased the basal activated cleaved Notch-1 (Val 1744) expression in PANC-1 cells but not in CD44^+^/CD24^+^spheres (Figure 6(a)).

In order to examine the biological relevance of decreased IGF-1R expression, IGF-1R expression was inhibited in PANC-1 cells utilizing pGIPZ-lentiviral-shRNA-IGF-1R expression vector (Figure 6(d)). Decreased IGF-1R expression inhibited the growth of the PANC-1 pancreatic cancer cells and increased the 3-Cl-AHPC-mediated inhibition of CD44^+^/CD24^+^sphere size (Figure 6(c) and Supplementary Figure S6A). Decreased IGF-1R expression in IGF1R-KD1 and IGF1R-KD2 cells significantly inhibited sphere formation by the PANC-1 CD44^+^/CD24^+^ cells and enhanced ARR induction of apoptosis in the IGF-1R knockdown PANC-1 cells (Figures 6(c) and 6(d)).

**Figure 7:**
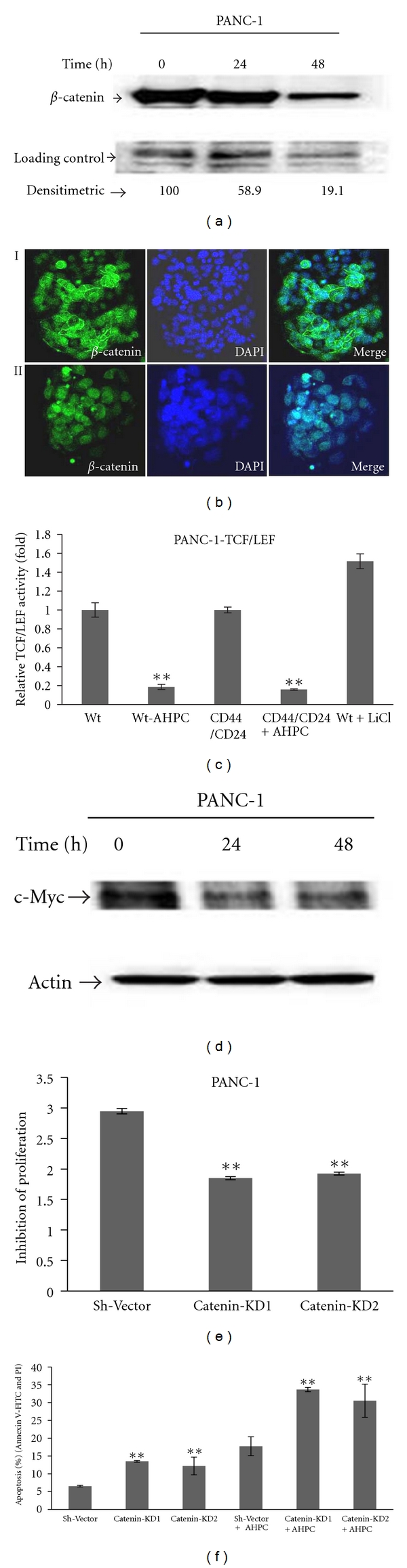
3-Cl-AHPC mediated inhibition of the activation of TCF/LEF in Wnt/*β*-catenin pathway and decreased of *β*-catenin nuclear localization. (a) 3-Cl-AHPC decreased nuclear *β*-catenin as indicated by Western blot using nuclear extracts and densitometric quantification. (b) Nuclear *β*-catenin in control- (i) and 3-Cl-AHPC- (ii) treated PANC-1 cells using confocal fluorescent microscope (magnification 40X). Cells were grown in eight chambered slides and then treated with 3-Cl-AHPC for 24 h. Slide was prepared as described in Section 2. DAPI was used for nuclear staining for 1 min and mounted the slide with prolong gold antifade kit. (c) 3-Cl-AHPC inhibited TCF/LEF activity in Wnt/*β*-catenin signaling in stably transfected Cignal TCF/LEF-Luc reporter PANC-1 cell lines and 50 mM LiCl was used as a positive control. For CD44/CD24 cells, TCF/LEF stably transfected cells were sorted by flow cytometry and followed the procedure same as wild type (Wt) stable cell line. Luciferase promoter activity values are expressed as fold using a total protein concentration for internal normalization. The error bars represent the mean of three separate determinations ± the standard deviation (SD). (d) 3-Cl-AHPC decreased Wnt/*β*-catenin signaling responsive c-Myc protein. ((e) and (f)) Knock down of *β*-catenin inhibited cell proliferations and enhanced more apoptosis in sh-*β*-catenin knockdown (KD) PANC-1 cell lines. Proliferation inhibition was evaluated after 72 h of seeding the cells by MTT assay and expressed as absorbance measured at 570 nm. The error bars represent the mean of three separate determinations ± the standard deviation (SD). ** (<0.01) was significantly different in comparison between sh-vector and Catenin-KDl/Catenin-KD2 and also between sh-vector and catenin-KD1/Catenin-KD2 treated with 3-Cl-AHPC, respectively.

Wnt/*β*-catenin signaling pathway leads to dephosphorylation, stabilization, and nuclear translocation of *β*-catenin. Nuclear *β*-catenin forms a complex with TCF/LEF family transcription factors and acts as a coactivator to express target genes in canonical Wnt signaling pathway such as CCND1 and MYC [27, 29]. We found that exposure to the 3-Cl-AHPC resulted in a decrease in nuclear *β*-catenin (Figures 7(a) and 7(b)) and also significantly decreased the TCF/LEF- transcriptional activity in wild-type cells as well as CD44^+^/CD24^+^ stably transfected TCF/LEF-sorted cells (Figure 7(c)). 3-Cl-AHPC decreased the expression of Wnt/*β*-catenin pathway responsive cyclin D1 and c-Myc in the PANC-1 cells within 24 h (Figures 5(d) and 7(d)). Inhibition of *β*-catenin expression using sh-RNA *β*-catenin significantly inhibited cell growth and enhanced 3-Cl-AHPC-mediated induction of apoptosis (Figures 7(e) and 7(f) and Supplementary Figure S6C). Thus, *β*-catenin expression and its general antiapoptotic effect mediated through a number of the *β*-catenin target genes inhibit ARR apoptosis induction.

## 5. Discussion

ARRs at physiologically achievable concentrations induce apoptosis of a number of pancreatic cancer cell lines as well as inhibit sphere formation by the CD44^+^/CD24^+^ stem-like cell population derived from the pancreatic cancer cell lines. Although ARRs were initially synthesized to demonstrate selectivity in the activation of retinoid nuclear receptor (RAR) subtypes, they have been shown to inhibit growth and induce apoptosis in different malignant cell types independent of RAR and retinoid x receptor (RXR) activation and function [30–34]. We found that Ras wild type and mutant pancreatic cancer cell lines COLO357, PANC-1, Capan-2, AsPc-1 cells, and MiaPaCa-2 display significant sensitivity to AHP3- and 3-Cl-AHPC-mediated growth inhibition and apoptosis induction.

Numerous mechanisms have been suggested through which ARR induces apoptosis in these cells [29–33]. The ability of the ARRs to enhance or inhibit the expression of a number of genes and proteins has been demonstrated [30–35]. We have found that both 3-Cl-AHPC and AHP3 significantly decreased IGF-1R and *β*-catenin expression and that the decreased expression of IGF-1R and *β*-catenin inhibited the growth and enhanced apoptosis of the pancreatic cancer cells suggesting that decreased IGF-1R and *β*-catenin expression potentiates ARR-mediated apoptosis.

Recent studies have demonstrated that malignant tumors are heterogeneous in composition with the stem cell population representing those cells that display resistance to chemotherapy and have greater metastatic potential [3, 29, 36]. Huang et al. utilized CD44/CD24/EpCAM positivity to identify the stem cell population in the PANC-1 pancreatic cell line [37]. They found that this stem cell population—which represented 2.1–3.5% of the total cell population—displayed a slower growth rate than CD44/CD24 negative cells and when injected rapidly formed large tumors in nude mice at week 4 while CD44/CD24 negative cells did not form tumors. Simeone similarly used CD24/CD44/EpCAM to identify a pancreatic CSC population that displayed the ability to form tumor cell spheres as well as enhanced tumor formation in nude mice [38]. In contrast, Hermann and colleagues utilized CD133 positivity to select a pancreatic cancer stem cell population from freshly isolated patient-derived tumors [3]. These CD133 positive cancer stem cells represented a heterogeneous population of tumor-initiating cells and only 500 of these cells were required to form orthotropic tumors in athymic mice [3]. Thus, markers purportedly in cancer stem cells appear not to detect all cancer stem cells in a particular tumor. We found however that spheres generated from CD44^+^/CD24^+^expressing pancreatic cancer cells also expressed CD133 as well as EpCAM. More recently, other markers, such as Aldehyde dehydrogenase (ALDH), have been found to be associated with the pancreatic cancer stem cell population [39].

IGF-1 and its receptor IGF-1R play a major role in proliferation, invasive potential, and metastatic behavior of pancreatic cancer cells [24–26]. IGF-1 exposure decreased phosphorylation and inactivation of PTEN and activation of PI3K, AKT, and the NF-*κ*B pathway in a number of pancreatic cancer cell lines resulting in their enhanced proliferation and invasiveness [24]. Other investigators have demonstrated that IGF-1R regulated hypoxia-inducible factor-1*α*, vascular endothelial growth factor, and angiogenesis through an autocrine loop in pancreatic cancer cells [25]. Further studies have documented that IGF-1/IGF-1R-mediated enhanced pancreatic carcinoma proliferation and invasiveness requires an interaction between IGF-1R and the hepatocyte growth receptor c-Met [26]. Dallas et al. have recently demonstrated enhanced IGF1R expression as well cancer stem cell markers in colon cancer cells displaying chemotherapy resistance [40]. In addition, these cells displayed greater sensitivity in terms of inhibition of growth following exposure to IGF-1R inhibitory antibody [40]. The addition of either ARR to the PANC-1, Capan-2, or MiaPaCa-2 cells downregulated expression of IGF-1R, cyclin D1, and *β*-catenin; decreased expression of these important proteins in the adherent cells, and the CD44^+^/CD24^+^ stem-like cells occurred prior to the onset of inhibition of cellular proliferation and the induction of apoptosis. We found that IGF-1R was significantly expressed in the pancreatic cancer sphere cells and its expression was markedly inhibited by exposure to 3-Cl-AHPC. The importance of IGF-1R in the proliferation of the pancreatic cancer cells, as well as their resistance to apoptosis, was documented by the observation that IGF-1R knockdown inhibited proliferation, enhanced 3-Cl-AHPC-mediated apoptosis, and inhibited sphere formation in PANC-1 cells.

Several investigators have demonstrated that *β*-catenin is essential for normal pancreatic development through the canonical Wnt signaling pathway but this pathway is downregulated in adult pancreas [41, 42]. Mutations in *β*-catenin or abnormal canonical Wnt signaling activity have been documented in pancreatic cancer [43–45]. Heiser et al. [45] have demonstrated that enhanced Wnt/*β*-catenin signaling in itself can induce pancreatic tumorgenesis and that activation of other oncogenes in the presence of enhanced Wnt/*β*-catenin signaling induces distinct pancreatic tumor formation. Addition of 3-Cl-AHPC downregulated *β*-catenin expression in pancreatic cancer cells and inhibited Wnt/*β*-catenin activation of transcription factor TCF/LEF and also downregulated Wnt/*β*-catenin pathway responsive genes cyclin D1 and c-Myc in PANC-1 cells. We found that *β*-catenin was significantly expressed in the pancreatic cancer sphere cells and that 3-Cl-AHPC inhibited this expression. 3-Cl-AHPC also downregulated GLI1, GLI2, and Ptch1 mRNA expression in the hedgehog pathway. ARR-mediated inhibitory effect on the self-renewal pathways, hedgehog and Wnt/*β*-catenin, may contribute to the inhibition of pancreatic cancer stem-like cell spheres. AHP3 and 3-Cl-AHPC may have a potential therapeutic role in the treatment of pancreatic cancer and further studies will delineate underlying mechanisms of inhibition for cancer stem cell self-renewal pathways.

## 6. Conclusions

Pancreatic cancer is resistant to chemotherapy and is a leading cause of cancer death. The adamantyl-substituted retinoid-related compounds 3-Cl-AHPC and AHP3 inhibit both pancreatic cancer and pancreatic stem-like cancer cells growth at physiologically achievable concentration. Inhibition of IGF-1R and *β*-catenin potentiates ARR-mediated growth inhibition and induction of apoptosis. 3-Cl-AHPC and AHP3 apoptosis induction in pancreatic cancer and pancreatic stem-like cancer cells suggested a potential therapeutic agent for pancreatic cancer.

## Supplementary Material

The CD44^+^/CD24^+^ cells were sorted by flow cytometry and approximately 200-300 cells were seeded with B27 medium in 96 well low attachment plates and 1 mM AHP3 added either the day after seeding or 7 days following spheroid formation. The sizes of spheroids were photographed and measured on a 100 mm scale and magnification 400 X using Olympus fluorescence microscope digital camera software and DP2-BSW software.
