# Supplementary material for: Adamantyl Retinoid-Related Molecules Induce Apoptosis in Pancreatic Cancer Cells by Inhibiting IGF-1R and Wnt/β-Catenin Pathways
Source: J Oncol. 2012 Apr 5;2012:796729. doi: 10.1155/2012/796729 (PMC3335256; doi:10.1155/2012/796729)
Supplement: Supplementary file 1 — The CD44+/CD24+ cells were sorted by flow cytometry and approximately 200-300 cells were seeded with B27 medium in 96 well low attachment plates and 1 mM AHP3 added either the day after seeding or 7 days following spheroid formation. The sizes of spheroids were photographed and measured on a 100 mm scale and magnification 400 X using Olympus fluorescence microscope digital camera software and DP2-BSW software. [file 796729.f1.pdf]

A

AHP3 added the day after seeding the cells

Cells-0 day | Spheres-7 days | Spheres-14 days | AHP3 added for 7 days (cells) | AHP3 added for 14 days (cells)

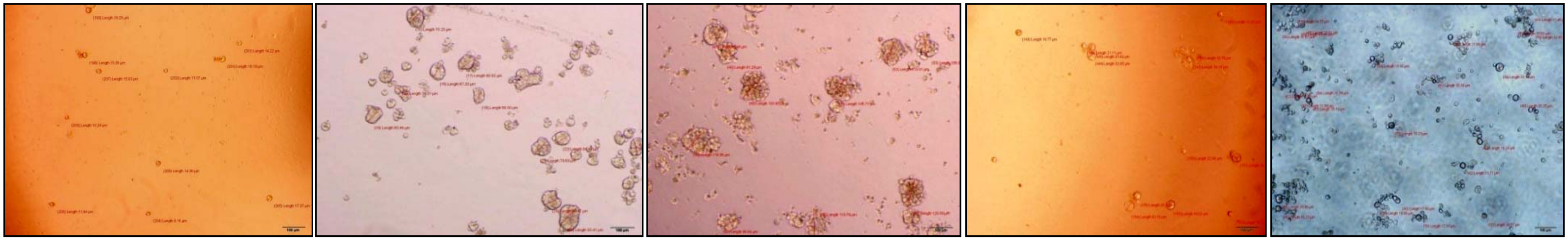

B

AHP3 added after spheres formation

Cells-0 day | Spheres-7 days | Spheres 14-Days | Spheroids at Day 7 prior to AHP3 addion | AHP3 added for 7 days

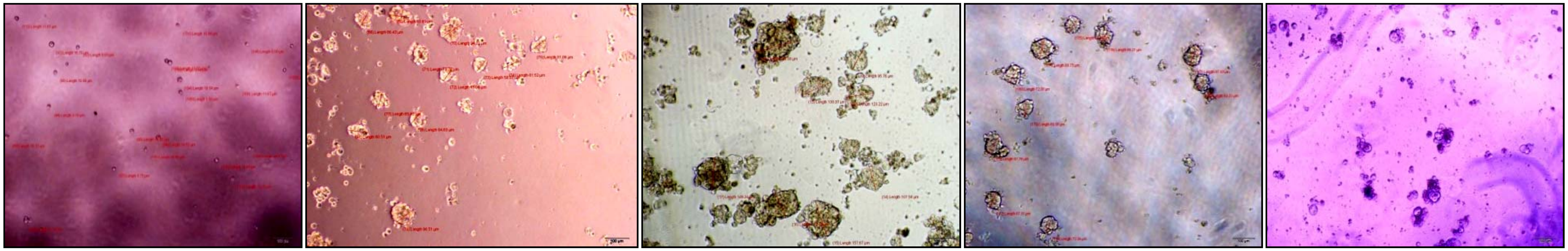

Supplementary Figure S1: CD44^{+}/CD24^{+} cell sphere formation and AHP3-mediated inhibition and degradation of spheres derived from PANC-1 cells (A) AHP3 inhibited CD44^{+}/CD24^{+} sphere formation in PANC-1 cells. (B) AHP3 exposure resulted in inhibition of growth and degradation of the CD44^{+}/CD24^{+} derived spheres in PANC-1 cells.

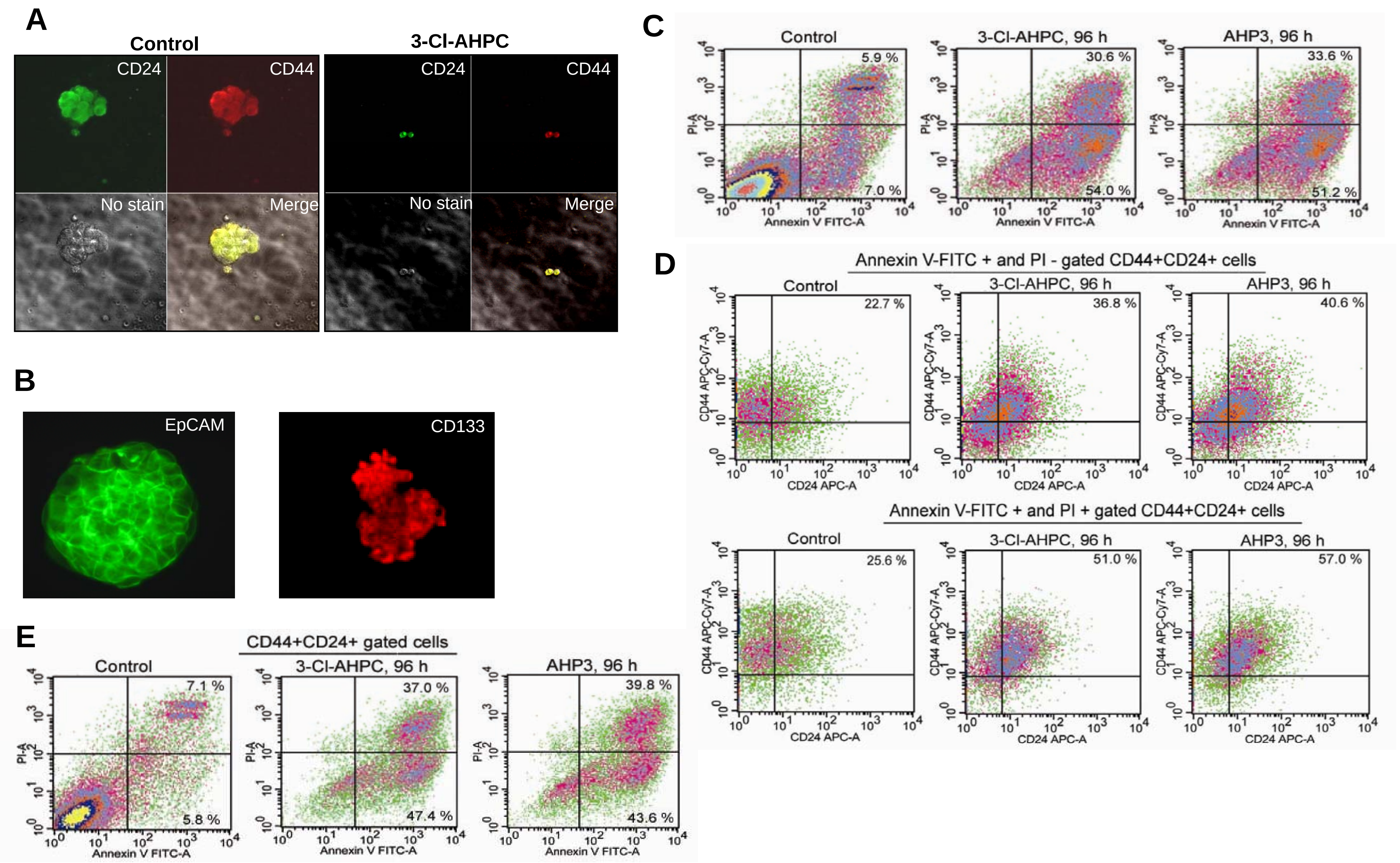

Supplementary Figure S2: Pancreatic spheres are composed of CD44$^+$/CD24$^+$/CD133$^+$/EpCAM$^+$ in cells and 3-Cl-AHPC (1 μM) mediated apoptosis of CD44/CD24 PANC-1 cells. (A) CD44/CD24 spheres were stained with CD44, CD24. Cells in vehicle and 3-Cl-AHPC (1μM) treated spheres for 7 days were stained with either CD44 or CD24. Images were obtained using confocal microscope and the images merged. (B) CD44/CD24 spheres expressed EpCAM and CD133 positivity by confocal and fluorescence microscope . (C) ARR-mediated total apoptotic cells (D) early apoptotic CD44$^+$/CD24$^+$ cells (upper panel) and early & late apoptotic CD44$^+$/CD24$^+$ cells (lower panel), and (E) total CD44$^+$/CD24$^+$ apoptotic cells. PANC-1 cells were analysed in flow cytometry.

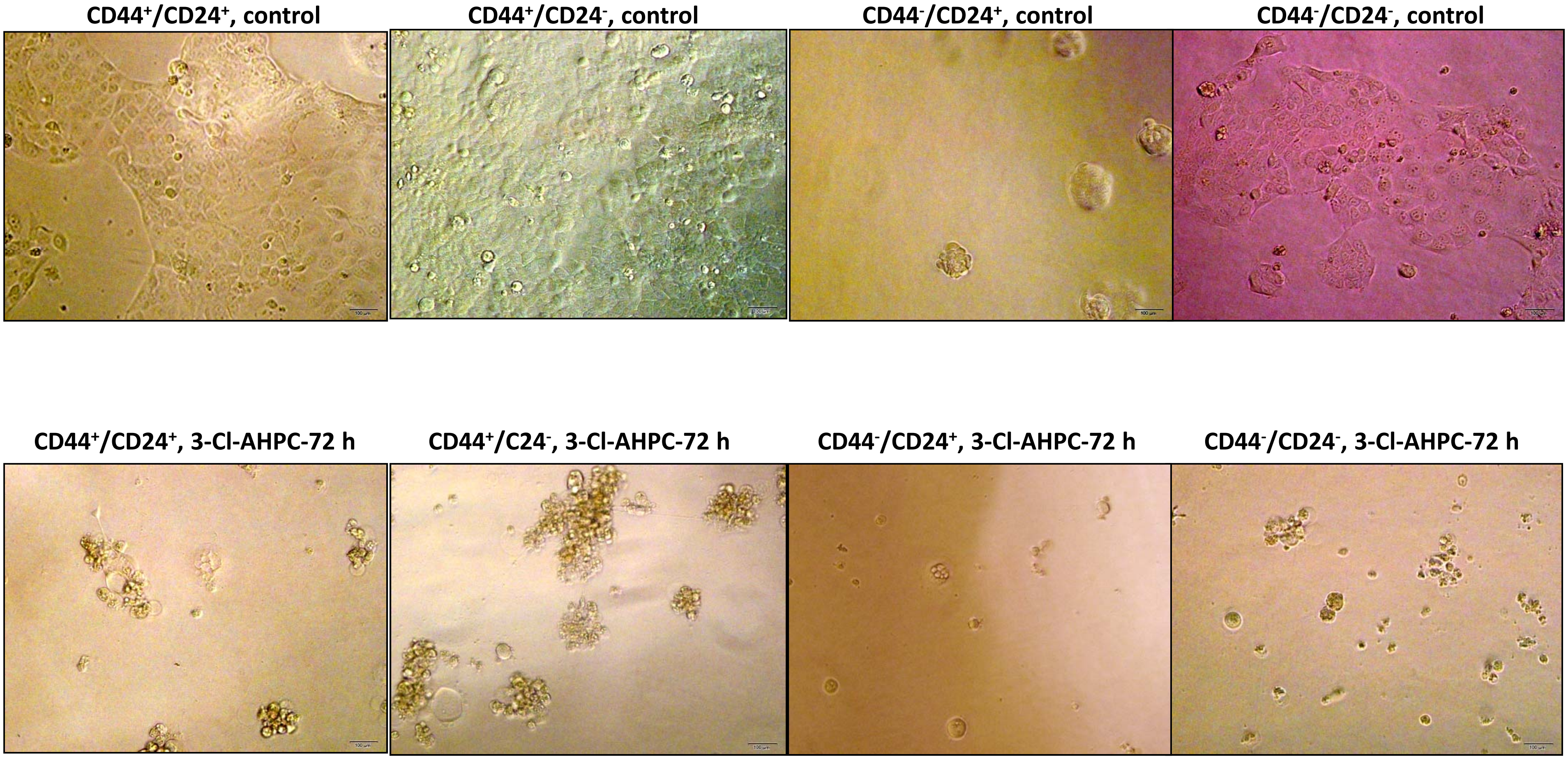

Supplementary Figure S3: 3-Cl-AHPC (1 μM) mediated growth inhibition and degradation of CD44/CD24 PANC-1 cells. The four populations of CD44/CD24 cells were sorted by flow cytometry and grown with B27 medium without growth factors in 24 well plates and 1.0 μM 3-Cl-AHPC added the day after seeding. The cells were photographed using Olympus microscope digital camera and DP2-BSW software.

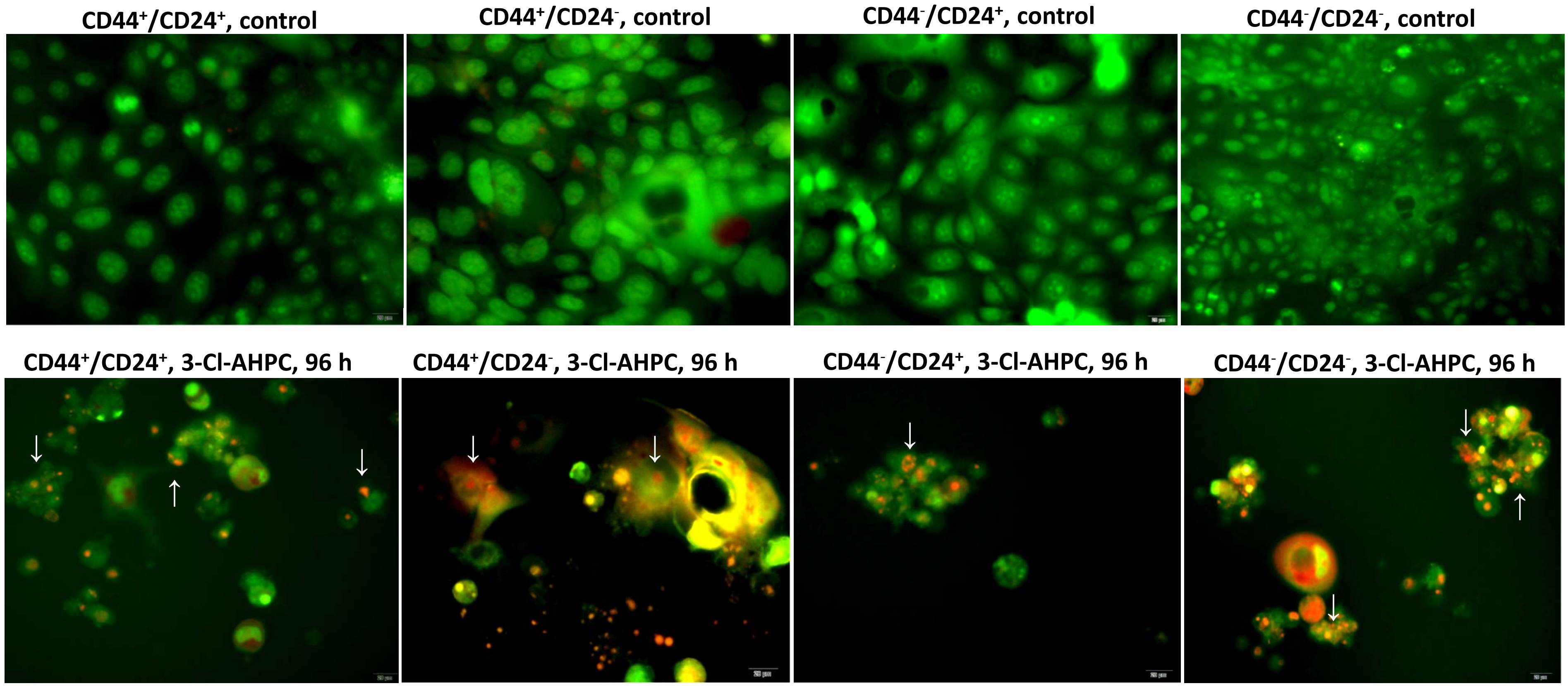

Supplementary Figure S4: 3-Cl-AHPC -mediated apoptosis of PANC-1 CD44/CD24 cell populations. The four types of CD44/CD24 cell populations were sorted by flow cytometry and were seeded with B27 medium without growth factors in 24-well plates. 1.0 μM 3-Cl-AHPC was added the day after seeding. Cells were stained with acridine orange/ethidium bromide. Arrow indicates fragmentation of DNA in apoptotic cells. The cells were photographed using Olympus microscope digital camera and DP2-BSW software.

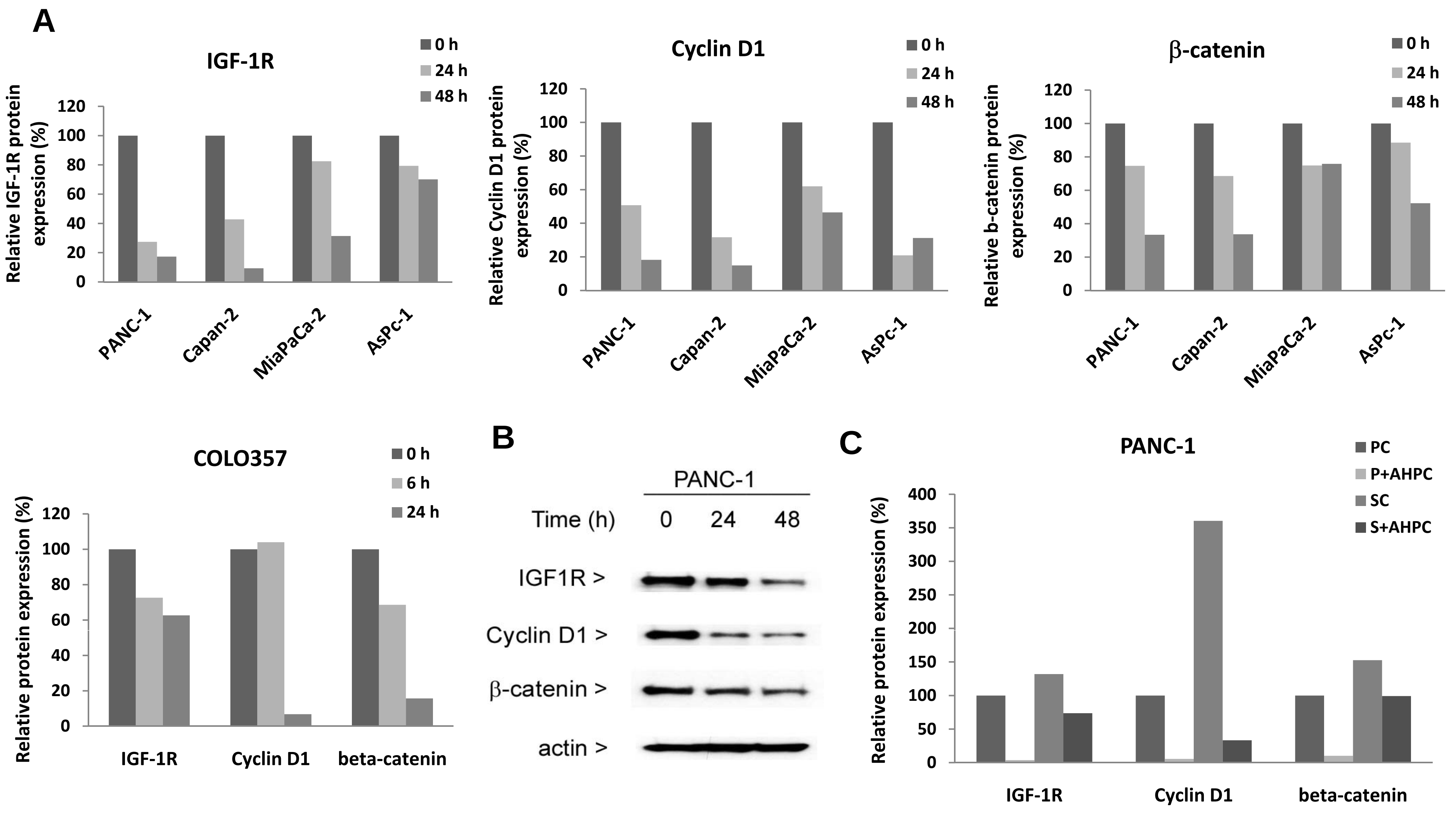

Supplementary Figure S5: (A) Densitometric analysis of protein expression of IGF-1R, cyclin D1 and β-catenin in pancreatic cancer cell lines. Cells were incubated with 1.0 μM 3-Cl-AHPC for times indicated and western blots performed as described in Materials and Methods section. (B) IGF-1R, cyclin D1 and β-catenin expression decreased in cells following exposure of AHP3. (C) Densitometric analysis of expression of IGF-1R, cyclin D1 and β-catenin in PANC-1 parental and spheroid cells. PC, parental control; SC, spheroid control; AHPC, 3-Cl-AHPC ; P+AHPC, parental cells 3-Cl-AHPC treated; S+AHPC, spheroid 3-Cl-AHPC treated. Parental cells and spheroids were treated for 96 h and 7 days, respectively.

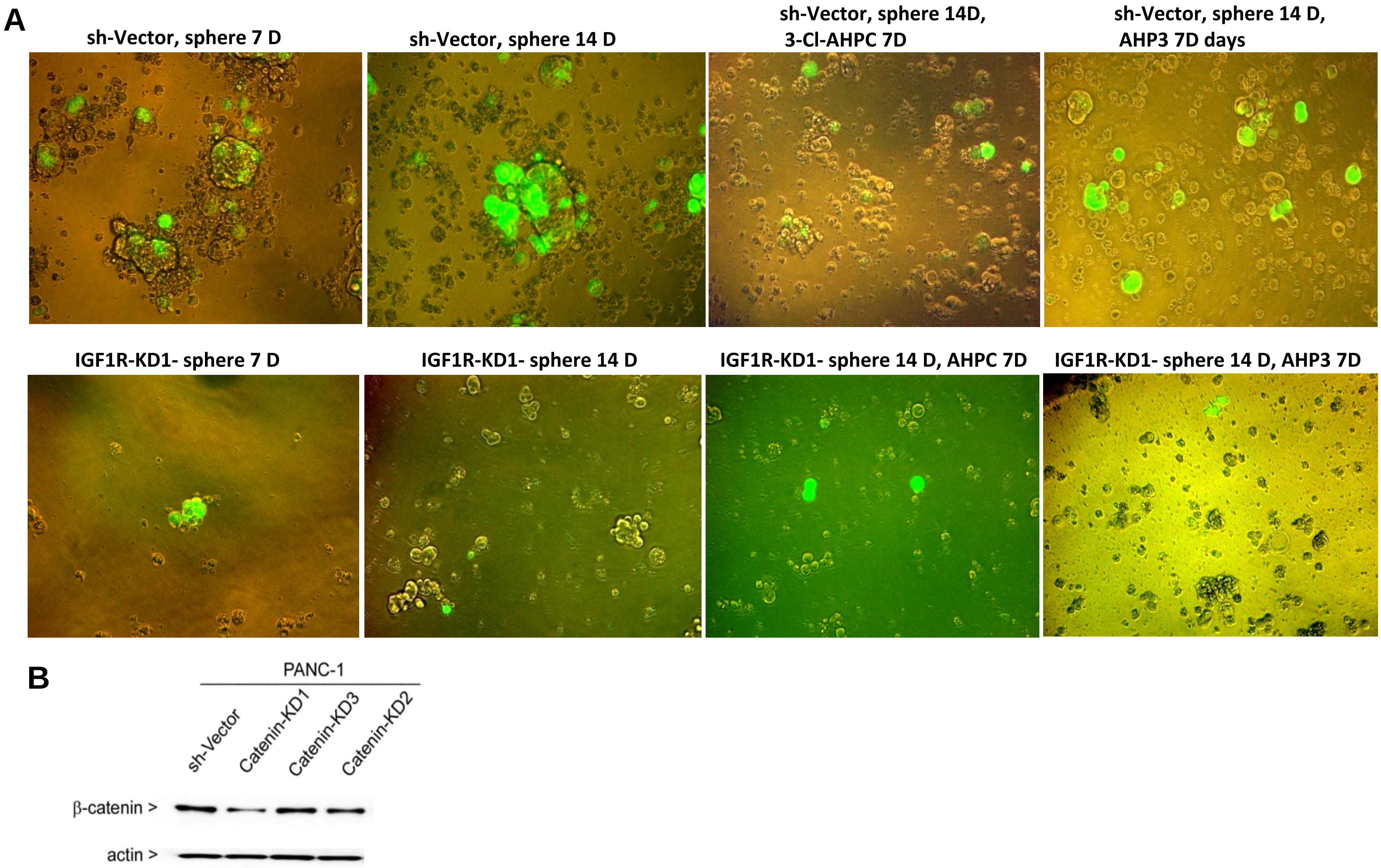

Supplementary Figure S6. (A) Knock down of IGF-1R expression inhibits PANC-1 sphere formation. Spheres were formed at indicated times (D, day) from stably transfected GFP-tagged sh-vector and GFP-tagged -IGF-1R-KD PANC-1 cells. Spheres were grown as described in Materials and Methods. Vehicle or 1 µM 3-Cl-AHPC or AHP3 were added after 7 days of sphere formation and the spheres incubated an addition 7 days. Spheres were visualized and photographed with fluorescence microscope (100 µm scale and magnification 400 X) after 14 days 3-Cl-AHPC treatment. (B) Knock down of β-catenin in stably transfected PANC-1 cell lines.
